# Supplementary material for: The Evolution of Mass Cell Suicide in Bacterial Warfare
Source: Curr Biol. 2020 Jul 20;30(14):2836–2843.e3. doi: 10.1016/j.cub.2020.05.007 (PMC7372221; doi:10.1016/j.cub.2020.05.007)
Supplement: Document S1. Figures S1 and S2 [file mmc1.pdf]

Current Biology, Volume *30*

Supplemental Information

The Evolution of Mass Cell Suicide in Bacterial Warfare

Elisa T. Granato and Kevin R. Foster

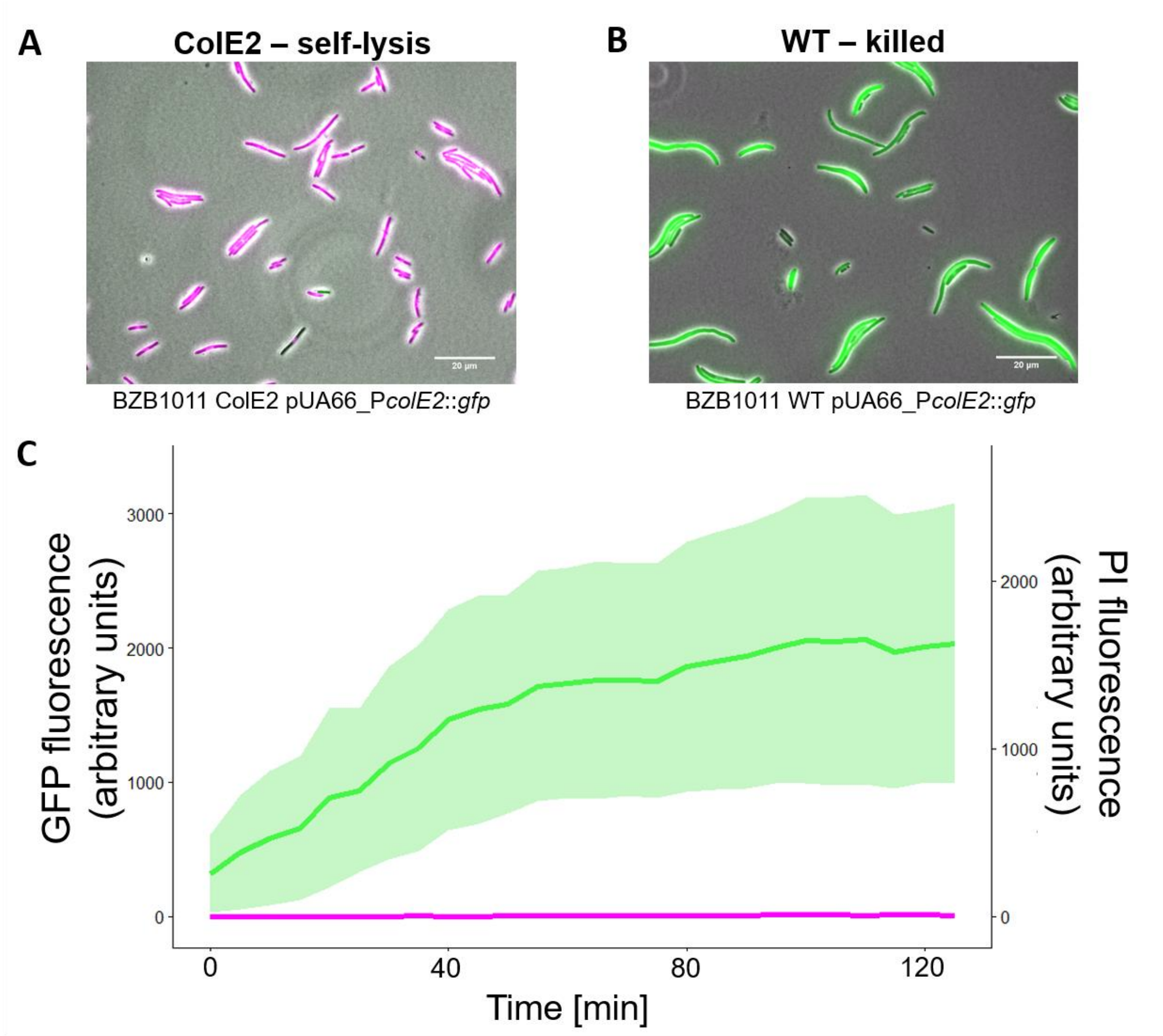

Figure S1. ***E. coli*** **cells unable to self-lyse do not exhibit elevated PI-fluorescence. See also Figure 2.** **(A+B)** Representative images of ColE2 pUA66-P*colE2*::*gfp* cells having undergone self-lysis **(A)** or WT pUA66-P*colE2*::*gfp* cells having been killed by a foreign DNase colicin **(B)**. Phase-contrast channel, GFP channel and propidium iodide (PI) channel are overlaid in all images. GFP signal indicates colicin promoter activation. PI signal indicates membrane permeabilization, i.e. self-lysis. Absence of PI signal in a non-dividing, dead cell is indicative of an intact membrane and hence killing by the action of the foreign DNase colicin. Image (A) represents the final timepoint of the dataset shown in Figure 2C. Image shown in (B) represents the final timepoint of the dataset shown in (C) here. Scale bars, 20 µm. **(C)** Fluorescence signals in WT cells responding to colicin E8. WT pUA66-P*colE2*::*gfp* cells were exposed to a 1% dilution of supernatant of a colicin E8-producing strain and imaged for up to 6 hours. Individual cell fluorescence tracks are shown for the GFP channel (green) and PI channel (magenta). Thick lines and shaded areas indicate the mean and standard deviation across n = 31 tracked cells in the same field of view. See Video S4.

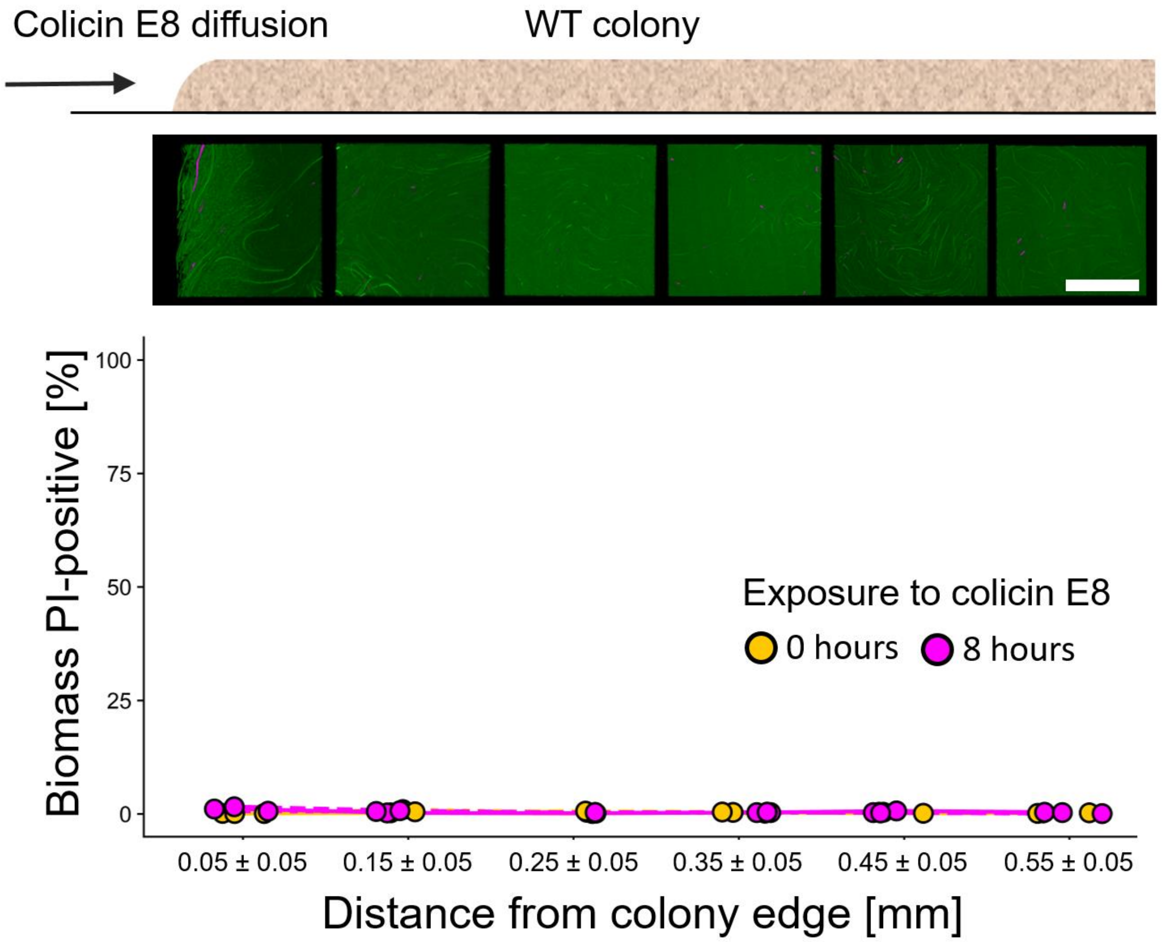

Figure S2. Three-dimensional *E. coli* colonies unable to self-lyse do not exhibit elevated PI-fluorescence. See also Figure 3. Quantification of PI-specific fluorescence in three-dimensional colonies exposed to colicin E8 produced by a nearby colony. Cells of a focal strain producing GFP constitutively but not capable of producing colicin E2 or self-lysing (WT *gfp*) were grown in a three-dimensional colony on nutrient medium supplement with propidium iodide (PI, 1µg/mL) next to a strain producing colicin E8. The focal colony was imaged for 8 hours using time-lapse 3D confocal microscopy at six locations situated at different distances from the colony edge facing the competitor. The proportion of PI-specific fluorescence at each location was quantified by determining the volume of biomass exhibiting PI-specific fluorescence relative to the total biomass, after zero or 8 hours of exposure to colicin E8. Total biomass was calculated by determining the volume of biomass exhibiting GFP-specific fluorescence (indicating either live cells or dead cells) plus biomass of those exhibiting PI-specific fluorescence. Line-types indicate three independent biological replicates. We did not detect a statistically significant increase in PI-specific fluorescence over the observation period at any location (linear model: percent.positive ~ timepoint; $F(1,4) \leq 11.89$ for all distances; $p > .01$). Images above each distance point show 3D renderings of confocal images of the same replicate colony acquired at the respective position after 8 hours of exposure, viewed from above. Scale bar, 50 µm.
