# Supplementary material for: The Evolution of Mass Cell Suicide in Bacterial Warfare
Source: Curr Biol. 2020 Jul 20;30(14):2836–2843.e3. doi: 10.1016/j.cub.2020.05.007 (PMC7372221; doi:10.1016/j.cub.2020.05.007)
Supplement: Document S2. Article plus Supplemental Information [file mmc8.pdf]

Current Biology

Report

The Evolution of Mass Cell Suicide in Bacterial Warfare

Graphical Abstract

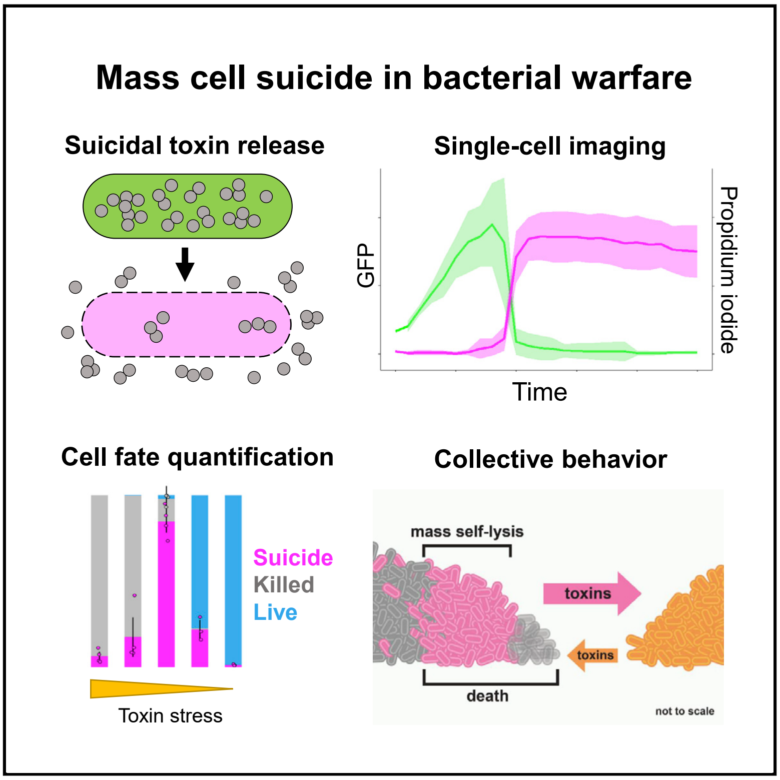

Highlights

- A novel assay can detect *Escherichia coli* undergoing cell suicide to release toxins
- This assay was used to follow the frequency of suicidal self-lysis during competition
- Under some conditions, nearly all cells will self-lyse to release toxins
- Self-lysis makes evolutionary sense as cells will die anyway from competitors' toxins

Authors

Elisa T. Granato, Kevin R. Foster

Correspondence

elisa.granato@zoo.ox.ac.uk (E.T.G.),
kevin.foster@zoo.ox.ac.uk (K.R.F.)

In Brief

Granato and Foster show that *Escherichia coli* bacteria undergo mass cell suicide to release toxins during competitions and that this behavior is restricted to cells that are going to die from the competitor's attack. Mass cell suicide makes evolutionary sense as a last-ditch attempt to counterattack by doomed cells.

Granato & Foster, 2020, Current Biology *30*, 2836–2843
July 20, 2020 © 2020 The Author(s). Published by Elsevier Inc.
https://doi.org/10.1016/j.cub.2020.05.007

CellPress
OPEN ACCESS

Current Biology

Report

The Evolution of Mass Cell Suicide in Bacterial Warfare

Elisa T. Granato[1,2,*] and Kevin R. Foster[1,2,3,*]
[1]Department of Zoology, University of Oxford, 11a Mansfield Road, OX1 3SZ Oxford, UK
[2]Department of Biochemistry, University of Oxford, 3 South Parks Road, OX1 3QU Oxford, UK
[3]Lead Contact
*Correspondence: elisa.granato@zoo.ox.ac.uk (E.T.G.), kevin.foster@zoo.ox.ac.uk (K.R.F.)
https://doi.org/10.1016/j.cub.2020.05.007

SUMMARY

Behaviors that cause the death of an actor are typically strongly disfavored by natural selection, and yet many bacteria undergo cell lysis to release anti-competitor toxins [1–5]. This behavior is most easily explained if only a small proportion of cells die to release toxins and help their clonemates, but the frequency of cells that actually lyse during bacterial warfare is unknown. The challenge is finding a way to distinguish cells that have undergone programmed suicide from those that were simply killed by a competitor's toxin. We developed a two-color fluorescence reporter assay in *Escherichia coli* to overcome this problem. This revealed conditions where nearly all cells undergo programmed lysis. Specifically, adding a DNA-damaging toxin (DNase colicin) from another strain induced mass cell suicide where ~85% of cells lysed to release their own toxins. Time-lapse 3D confocal microscopy showed that self-lysis occurs locally at even higher frequencies (~94%) at the interface between toxin-producing colonies. By exposing *E. coli* that do not perform lysis to the DNase colicin, we found that mass lysis occurs when cells are going to die anyway from toxin exposure. From an evolutionary perspective, this renders the behavior cost-free as these cells have zero reproductive potential. This helps to explain how mass cell suicide can evolve, as any small benefit to surviving clonemates can lead to this retaliatory strategy being favored by natural selection. Our findings have parallels to the suicidal attacks of social insects [6–9], which are also performed by individuals with low reproductive potential.

RESULTS

E. coli and many other bacterial species release large protein toxins, often known as bacteriocins, from their cells via cell lysis [1–5]. To study the extent of cell suicide during bacterial competition with these toxins, we focused on the well-studied group A colicins in *E. coli*. These are expressed from small, medium-copy plasmids and function to kill closely related strains and species [1]. Producing cells permeabilize their own membrane with a dedicated lysis protein to release toxins into the environment, killing themselves in the process [1, 2, 10]. When a colicin-producing strain is growing alone, the colicin operon is typically only expressed in a small fraction of the population [11–17]. Expression can be upregulated by DNA damage as the colicin operon is regulated by the SOS response pathway [16, 18, 19], which is often done artificially via the addition of DNA-damaging agents such as mitomycin C [14, 16, 18, 20, 21]. However, many natural colicins also damage DNA, and these too have been shown to upregulate colicin production in targeted cells [11, 22]. Colicinogenic strains are thus capable of retaliation, whereby they release their own colicins upon exposure to the colicins of another strain, an example of competition sensing via regulatory responses to DNA damage (Figure 1) [23]. This raises the possibility that, in competition between *E. coli* strains using DNA-damaging colicins, there may be high levels of suicidal colicin release in the population. However, to assess this, one needs to be able to distinguish between cells that are undergoing cell suicide in response to sensing a competitor's DNA-damaging toxin and those that were killed by the toxin itself. Moreover, we reasoned that high frequencies of a lethal behavior would be both surprising and interesting from an evolutionary standpoint, and one that has few known precedents in the natural world. We therefore sought to develop a method that would allow us to make the critical distinction between a cell killing itself via an evolved behavior, and one simply dying due to exposure to a DNA-damaging agent.

Self-lysis Frequency Is Modulated by Competitor Toxin Concentrations

We followed the activation of the colicin E2 operon, encoding the toxin, its cognate immunity protein, and the lysis protein (Figure 1A), using a reporter plasmid that expresses green fluorescent protein (*gfp*) from the native colicin E2 promoter (pUA66-P*colE2*::*gfp*) [11]. Cells carrying this reporter widely upregulate GFP expression when exposed to DNA-damaging toxins made by a competitor (Figures 1B–1D). This construct, like others previously studied [12, 14, 24, 25], allows one to identify cells that may be on their way to cell suicide via lysis [1, 2, 10]. However, reporting promoter activation is not sufficient to follow the full behavior, as the self-lysis process is dependent on the

 © 2020 The Author(s). Published by Elsevier Inc.
This is an open access article under the CC BY license (http://creativecommons.org/licenses/by/4.0/).

CellPress
OPEN ACCESS

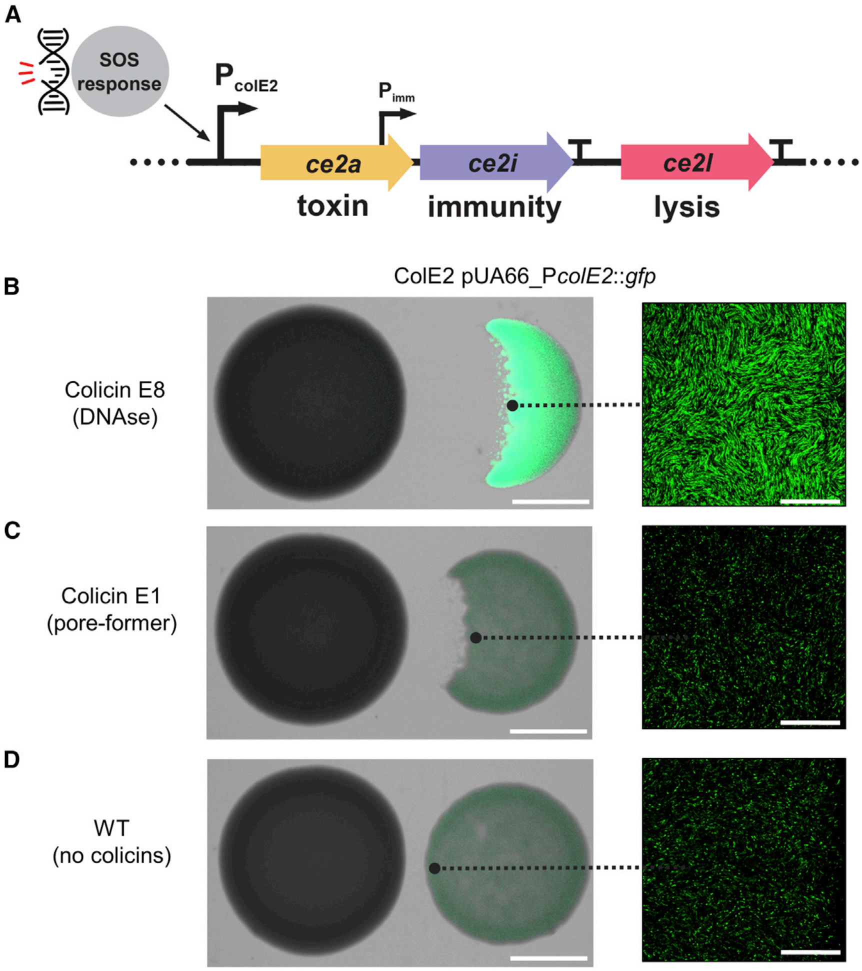

Figure 1. *E. coli* Activates the Colicin Operon in Response to a Competitor Causing DNA Damage

(A) Overview of the colicin operon as encoded on plasmid pColE2-P9 in *E. coli*. In response to DNA damage, the P_{colE2} promoter is activated and the genes encoding colicin E2 (*ce2a*) and its cognate immunity protein (*ce2i*) are expressed. To ensure sufficient levels of immunity protein, *ce2i* is additionally transcribed from a second, constitutively active promoter (P_{imm}) located within the *ce2a* gene. A transcriptional terminator (T) after the immunity gene *ce2i* ensures that the downstream gene encoding the lysis protein (*ce2l*) is only expressed at very high levels of P_{colE2} activation. Adapted from [1].

(B) The response of the *E. coli* colicin E2 promoter to a foreign DNase colicin (colicin E8). *E. coli* BZB1011 colonies producing colicin E2 were grown next to competitor colonies overnight and then imaged by stereomicroscopy (left) and confocal microscopy (right). In the colicin E2 producer, the colicin E2 promoter also drives the expression of GFP on a reporter plasmid (pUA66-P*colE2*::*gfp*).

(C) Absence of response of the *E. coli* colicin E2 promoter to the pore-forming colicin E1 made by *E. coli* BZB1011 carrying the colicin E1 plasmid.

(D) Wild-type control where the left-hand strain (BZB1011) lacks any colicin plasmid and so does not produce colicins. Scale bars, 2 mm (left) and 50 μm (right).

expression of the lysis gene in the colicin operon [20, 26], which is subject to several additional layers of regulation at both the transcriptional and post-transcriptional level [19, 27–30]. Moreover, with this reporter alone, it is impossible to tell whether a cell that died was killed by external toxins while building up colicins or if it had undergone self-lysis. We therefore sought to identify ways to distinguish the self-lysis phenotype from cell death and discovered that one of the standard DNA dyes used in microbiology—propidium iodide—reliably identified cells that have undergone self-lysis. Propidium iodide (hereafter PI) is typically used as a "dead" stain, because it will only enter cells with a compromised membrane and bind their DNA, generating a fluorescent signal [31]. We found that self-lysis leads to PI reliably entering cells to give the fluorescent signal, but, critically, cell death caused by the DNase colicin toxin of another *E. coli* strain does not. When combined with the fluorescent signal from the GFP reporter plasmid [11], cells then exhibit a characteristic two-color fluorescence pattern when undergoing self-lysis but not when they are killed by foreign DNase toxins (Figures 2A, 2B, and S1; Videos S1 and S2).

Using this assay, we set out to characterize the response of a colicinogenic strain to a competitor producing a foreign DNA-damaging colicin (E8), which is expected to elicit SOS response, colicin production, and subsequent self-lysis. We began by exposing the colicin E2-producing strain (ColE2) carrying the reporter plasmid (pUA66-P*colE2*::*gfp*) to sterile supernatants of a colicin E8-producing competitor on nutrient agar containing PI. Using time-lapse fluorescence microscopy and cell tracking, we measured GFP and PI fluorescence levels in thousands (n = 7,985) of individual cells exposed to colicin E8 at a range of concentrations (Figures 2C and 2D; Video S3). In cells undergoing self-lysis, the GFP signal steadily increased for approximately 50 min leading up to the lysis event, representative of the continuous activation of the colicin promoter in response to the imposed DNA damage and thus accumulation of colicins in the cytoplasm (Figure 2C). Self-lysis events were then characterized by the simultaneous influx of PI binding to DNA (increased PI fluorescence signal) and efflux of GFP out of the cell (decreasing GFP signal) due to leakage of the cytoplasm into the environment (Figure 2C).

We combined these fluorescence measurements with cell viability observations to divide individual cells' responses to colicin E8 into three categories (Figure 2D): non-dividing and PI-negative (killed cell) or PI-positive (self-lysed cell), as well as dividing and PI-negative (live cell). At the highest concentration of toxic supernatant, most cells were immediately killed upon exposure to colicin E8, and little self-lysis occurred (6.3%). In treatments with successively lower concentrations of colicin E8, however, the proportion of self-lysed cells increased dramatically as cells had more time to respond to the stress, reaching a peak at 1% supernatant concentration of 84.7% self-lysed cells

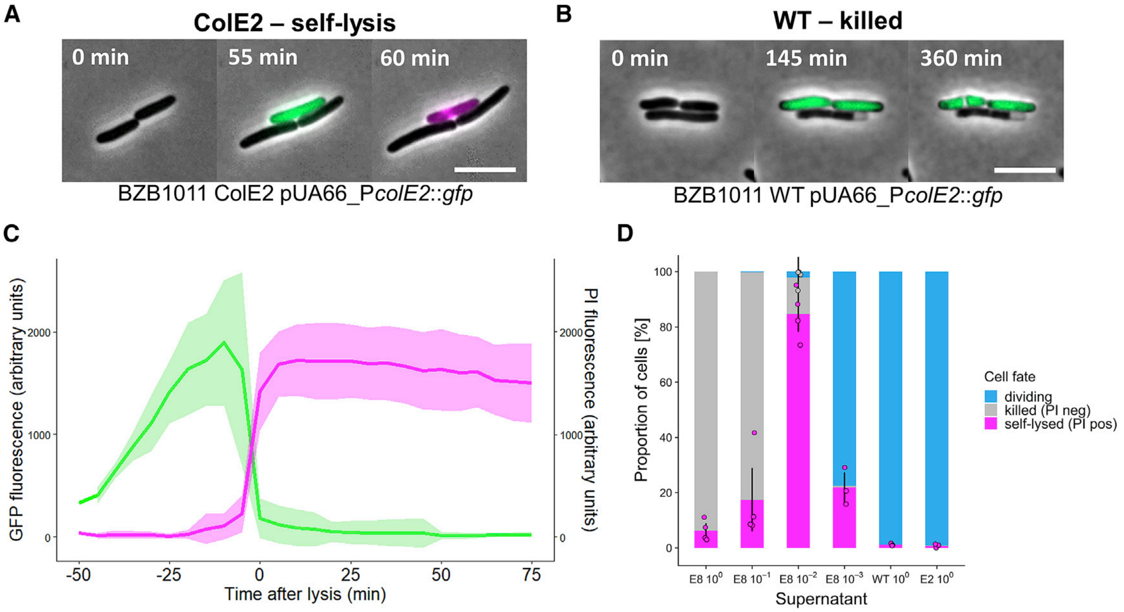

Figure 2. Self-lysis Frequency in *E. coli* Is Modulated by Competitor Toxin Concentrations

(A) Representative time-lapse micrographs of ColE2 pUA66-P*colE2*::*gfp* cells undergoing self-lysis. Phase-contrast channel, GFP channel, and propidium iodide (PI) channel are overlaid in all images. GFP signal indicates colicin promoter activation. PI signal indicates membrane permeabilization, i.e., self-lysis. Scale bar, 5 μm. See Video S1.

(B) Representative time-lapse micrographs of wild-type cells (WT pUA66-P*colE2*::*gfp*) being killed by a foreign DNase colicin (colicin E8). Phase-contrast channel, GFP channel, and propidium iodide (PI) channel are overlaid in all images. The absence of PI signal in a non-dividing, dead cell is indicative of an intact membrane and killing by the action of the colicin. Scale bar, 5 μm. See Video S2.

(C) Fluorescence signals in cells undergoing self-lysis in response to colicin E8. ColE2 pUA66-P*colE2*::*gfp* cells were exposed to a 1% dilution of supernatant of a colicin E8-producing strain and imaged for up to 6 h. Individual cell fluorescence signals are shown for the GFP channel (green) and PI channel (magenta). Thick lines and shaded areas indicate the mean and standard deviation across n = 20 lysed cells in the same field of view. See Video S3. Figure S1C and Video S4 show a negative control where colicin E8 is added to wild-type cells that lack the colicin plasmid.

(D) Cell-fate frequencies in populations of *E. coli* exposed to colicin E8. ColE2 pUA66-P*colE2*::*gfp* cells were exposed to different dilutions of supernatant of a colicin E8-producing strain, a non-producing wild-type, or their own sterile supernatant. Cells were imaged for up to 6 h, and the fate of n = 7,985 cells across all treatments was categorized as either dividing, killed (non-dividing and PI-negative), or self-lysed (non-diving and PI-positive). Error bars indicate SEM across three or four biological replicates. A Kruskal-Wallis test yielded a statistically significant relationship between supernatant concentrations and self-lysis frequencies (chi-square test = 18.285, df = 5, p = 0.0003).

on average, with a maximum value of up to 95% in one experiment (Figure 2D). At lower concentrations of toxin-containing supernatant, the self-lysis response became less frequent and more cells started to divide (21.9% self-lysis), which is expected as the amount of DNA damage experienced by the cells should be much lower. Taken together, these results show that colicinogenic cells respond to DNA-damaging competitor colicins with self-lysis in a concentration-dependent manner. Moreover, we found conditions where cells will undergo mass self-lysis with the great majority of cells dying to release toxins.

Mass Cell Suicide Is Also Seen in Competitions between *E. coli* Strains

Our first experiments showed that adding supernatant containing a DNase colicin results in a dose-dependent self-lysis response, with conditions where nearly all cells lyse. However, in nature, bacteria are likely to be exposed to toxin concentrations that change over both time and space as they compete with adjacent populations of cells [32, 33]. To capture these effects, we followed the lysis response in a monolayer colony of our focal strain (ColE2 pUA66-P*colE2*::*gfp*) with a colicin E8-producing competitor growing next to it (Video S5). In addition to allowing for toxin concentrations to change in time and space, this setup also allows the two strains to react to each other. In particular, both strains use DNase colicins that will activate the SOS response and trigger colicin production in the other strain, an example of competition sensing [11, 22, 23]. We seeded the colicin E2-producing strain carrying the reporter plasmid (ColE2 pUA66-P*colE2*::*gfp*) or a non-producing wild-type strain (WT pUA66-P*colE2*::*gfp*) in thin monolayer colonies on nutrient agar, with their colicin E8-producing competitor growing next to them. Using time-lapse fluorescence microscopy at the interface between the two colonies, we then tracked thousands (n = 3,420) of individual cells as they reacted to the incoming flow of colicin E8 toxins (Video S5) and measured self-lysis frequencies as a function of their distance from the interface (Figures 3A and 3B). As expected, in the wild-type control we observed that very few cells exhibited a PI fluorescence signal, consistent with PI being a good indicator of self-lysing cells. For the colicin E2-producer, we found that self-lysis frequencies within 6 h of observation were on average 75.5% at the colony edge and 97.3% in cells positioned slightly further (ca. 0.5 mm) away from the edge. For cells positioned even further away from the edge (>1.2 mm), self-lysis frequencies rapidly decreased to 0.8% (Figure 3B).

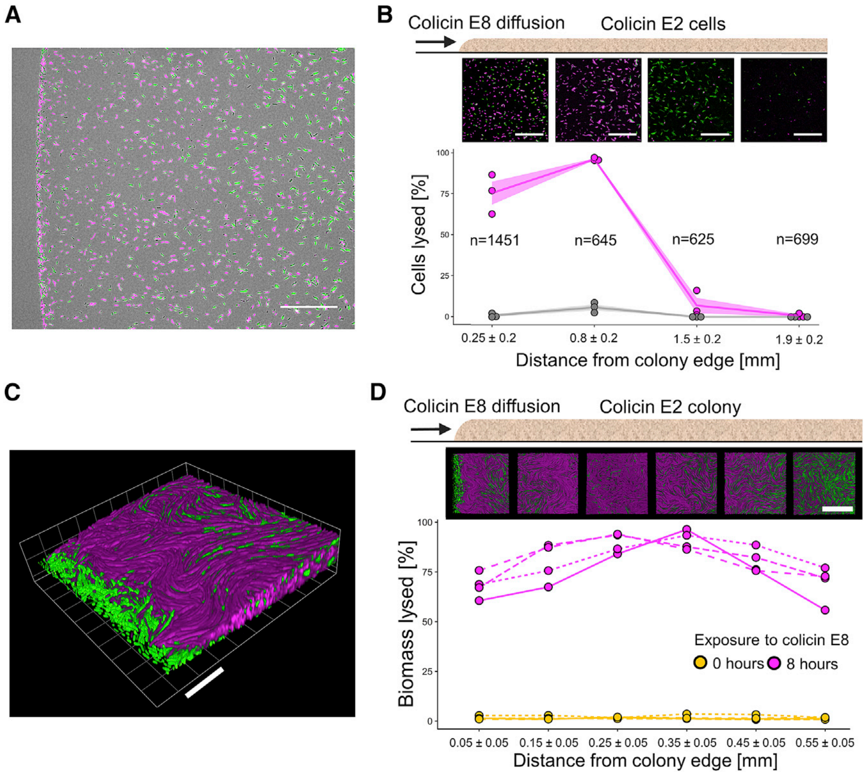

Figure 3. Colonies Facing a Competitor Exhibit Local Mass Self-lysis

Self-lysis quantification in colonies exposed to colicin E8 produced by a nearby colony.

(A and B) Cells of a strain either capable of producing colicin E2 and self-lysing (ColE2 pUA66-P*colE2*::*gfp*) or a wild-type non-producer incapable of self-lysis (WT pUA66-P*colE2*::*gfp*) were grown in monolayer colonies next to a ColE8 competitor. Cells were time-lapse imaged at different distances from the colony edge facing the competitor using fluorescence microscopy. (A) Representative image of a ColE2 pUA66-P*colE2*::*gfp* colony edge after 2 h of exposure to the competitor. Scale bar, 100 μm. (B) Self-lysis frequencies in ColE2 pUA66-P*colE2*::*gfp* cells (magenta) and WT pUA66-P*colE2*::*gfp* cells (gray) tracked over 6 h (n = cells tracked over ≥4 h). Lines and shaded areas indicate mean ± SEM across three biological replicates. Representative fluorescence images of ColE2 pUA66-P*colE2*::*gfp* cells after 4 h are shown above each distance point. Scale bar, 100 μm. Strains exhibited significantly different frequencies of propidium iodide (PI)-specific fluorescence at distances 0.25 and 0.8 mm (linear model: percent.lysis ~strain; $F(1,4) = 114.1$ for distance 0.25, 2,368 for distance 0.8; $p < 0.001$). See Video S5.

(C and D) Cells producing GFP constitutively and capable of producing colicin E2 and self-lysing (ColE2 *gfp*) were grown in three-dimensional colonies next to a ColE8 competitor. Colonies were time-lapse imaged for 8 h at different distances from the colony edge facing the competitor using 3D confocal microscopy. (C) A 3D-rendered image of a colony edge after 8 h of exposure to colicin E8 flowing in from the bottom-left corner of the image. Scale bar, 20 μm. (D) Self-lysis relative to total biomass before and after 8 h of exposure to the competitor. Line types indicate four biological replicates. Self-lysis frequency significantly increased during exposure (linear model: percent.lysis ~time point; $F(1,6) > 212.2$ for all distances; $p < 0.001$). Representative confocal images of a colony viewed from above after 8 h of observation are shown above each distance point. Scale bar, 50 μm. See Video S6. See Figure S2 for the same experiment using a wild-type, non-producing control strain.

This strong spatial relationship is likely explained by the competitor toxin diffusing toward and through the colony, leading to different micro-environments for cells in different locations. Consistent with this, at the colony edge, the cells that did not lyse failed to divide (Video S5), indicating that these cells experienced lethal stress levels that killed them so quickly that it prevented the self-lysis response. However, cells further into the colony were able to achieve mass self-lysis, presumably because the toxin built up more slowly at their more distant position, preventing immediate death and allowing for widespread self-lysis. As expected from this interpretation, we see more and more cell division at locations further into the colony, as cells get more time to grow and divide before being exposed to the toxin (data not shown). At this point, stress levels in a large fraction of the population failed to reach the critical threshold required for induction of the lysis gene, resulting in a strong decline in self-lysis frequency (Figure 3B). These results demonstrate that, when colicinogenic colonies are exposed to a competitor secreting a DNase toxin, self-lysis frequencies strongly depend on the distance from the competitor, and peak at intermediate distances, where cells are not immediately killed but still experience high enough stress levels to trigger self-lysis. They also suggest that the extremely high self-lysis frequencies observed in response to supernatants (Figure 2D) represent realistic behavioral patterns that can be recapitulated in spatially structured colonies facing a competitor (Figure 3B).

Bacteria often grow in dense biofilms or host-associated communities, where cell-cell interactions between clonemates—in addition to interactions between genotypes—can play a key role in their behaviors [32, 35–37]. In the case of colicins, the production of some DNase colicins—including colicin E2—can be induced by the presence of the same colicins released by clonemates, a phenomenon termed "autoinduction" [11, 17, 38]. We therefore sought to follow the lysis response in bacterial colonies at high cell density, where autoinduction is expected to be maximal. To image single-cell behavior at the interface between two dense bacterial communities, we used time-lapse 3D confocal microscopy, which allows for single-cell resolution of fluorescence signals over space and time. We followed a dense multilayer colony of colicin E2-producing cells constitutively producing GFP (ColE2 *gfp*), with a colicin E8-producing strain growing next to it (Video S6). This setup allowed us to measure self-lysis frequencies as the percentage of total biomass over time and as a function of their distance from the colony edge facing the competitor (Figures 3C and 3D). We found that within 8 h of observation 60.1%–75.8% of the total biomass self-lysed at the very edge of the colony (<0.1 mm), where many cells died from toxin exposure before self-lysing (Figure 3C, green area). Maximum self-lysis levels of 93.5%–96.4% was reached in locations further away from the edge (0.2–0.4 mm). For cells positioned even further away from the edge (>0.5 mm), self-lysis frequencies decreased again to 55.8%–77.0% (Figure 3D). This

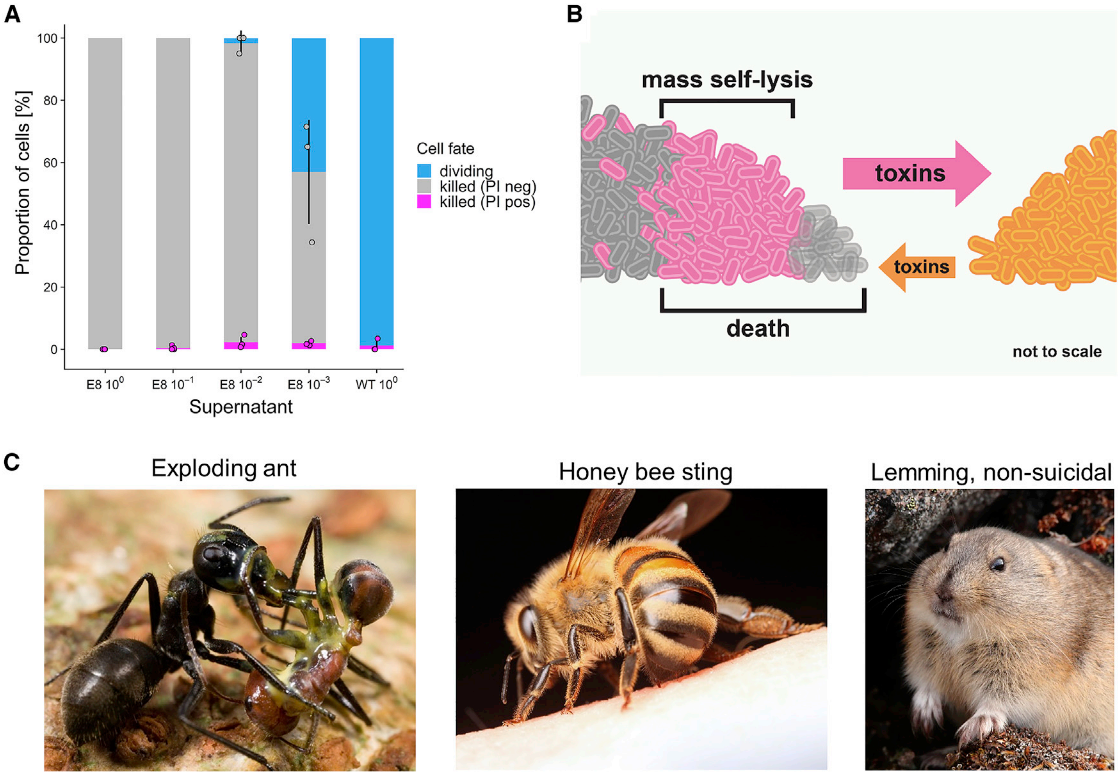

Figure 4. Suicidal Behavior Is Associated with Low Reproductive Potential

(A) Cell-fate frequencies in response to colicin E8 in *E. coli* cells unable to self-lyse. WT pUA66-P*colE2*::*gfp* cells were exposed to different dilutions of supernatant of a colicin E8-producing strain or a non-producing wild-type. Cells were imaged for up to 6 h, and the fate of n = 3,666 cells across all treatments was categorized as either dividing, dead and PI-negative, or dead and PI-positive. Error bars indicate SEM across three or four biological replicates. A Kruskal-Wallis test did not yield a statistically significant relationship between supernatant concentrations and frequency of cells positive for PI-specific fluorescence (chi-square test = 7.8486, df = 4, p = 0.09).

(B) Illustration of the mass cell suicide phenotype. In the region where the toxins (orange arrow) of the competing strain (orange) reaches lethal levels, large number of cells of the focal strain lyse (pink) and release colicins *en masse* (pink arrow). Cell suicide does not occur at the very edge of the colony on the left (transparent gray cells), as these cells die immediately from the competitors toxins.

(C) Two examples of suicidal behaviors in social insects, and a popular misconception. Left: a minor *Colobopsis cylindricus* ("exploding ant") worker has ruptured her body to release a sticky yellow substance, killing both herself and her opponent, the larger worker of another ant species (*Camponotus sp.*) [7, 34]. Middle: honeybee (*Apis mellifera*) workers sting in defense of their colony, which often results in the worker's death as the sting gets pulled out of her body [8]. Right: a long-standing myth incorrectly holds that lemmings commit mass suicide. The example is illustrative because—unlike in the social insects and bacteria where low reproductive potential and benefits to kin can explain suicidal behaviors—there is no evolutionary rationale for such behavior in lemmings. Image sources: ants (Mark Moffett/Minden Pictures, used with permission); honeybee (Alexander Wild, used with permission); northern collared lemming (Jeremy Gatten, used with permission).

spatial dependence of self-lysis frequencies mirrors our observations in thin monolayer colonies (Figure 3B) and is likely caused by some cells at the very edge dying before self-lysing, while cells further into the colony exhibit near-complete self-lysis. These results demonstrate that mass self-lysis also occurs in three-dimensional, high-density bacterial colonies in response to a competitor.

Mass Cell Suicide Is Associated with the Loss of Reproductive Potential

The discovery of high levels of cell suicide was initially surprising, given the typically strong natural selection against such individually costly behaviors. The evolutionary explanation for such behaviors requires that clonemates benefit from the behavior and that this benefit outweighs the cost of the dying cells, to maintain the trait in the population (an example of kin selection) [39–41]. The potential for these benefits became much clearer when we introduced spatial structure in the experiments and studied the cases where colicins diffuse into colonies from the side. Here, only the front line of cells engage in mass cell suicide with the potential of clonemates behind to benefit from their behavior (Figure 3). However, the proportion of cells that benefit in this way is likely to vary widely, dependent on the size and shape of competing bacterial groups, and other factors. Moreover, even with these benefits, it is striking to see so many cells engaging in a suicidal behavior, which removes nearly all cells from the edge of the colony and greatly slows expansion into new territory.

To understand the conditions of self-lysis better, therefore, we asked what would have happened to these cells had they not been able to self-lyse. We repeated the supernatant exposure experiment (Figure 2D) with a control strain that is genetically identical to the self-lysing strain except that it lacks the colicin

plasmid and thus the ability to produce colicins and self-lyse (WT pUA66-P*colE2*::*gfp*). This strain is still susceptible to colicin E8 and also carries the reporter plasmid (Figure 2B), which indicates whether the colicin promoter would be active if the cells were able to make colicins. We exposed this strain to supernatants containing colicin E8 at a range of concentrations and quantified the fate of the exposed cells (Figure 4A). Importantly, under conditions where we observed the highest levels of self-lysis in the colicinogenic strain (Figure 2D, third bar), we observed that the great majority of wild-type control cells were dead (Figure 4A, third bar). This indicates that the level of DNA damage these cells experienced when exposed to the DNase colicin E8 was too much to be repaired, resulting in their death. Therefore, the colicinogenic cells that self-lysed *en masse* under the same conditions (Figure 2D) most likely experienced a lethal level of stress imposed by the competitor toxin. The fitness cost of self-lysing is thus minimal, as the cells have low or no future reproductive potential.

DISCUSSION

We have identified competition scenarios where bacteria will engage in mass cell suicide, with the proportion of cells engaging in the behavior reaching almost 100% in some areas (Figure 4B). It is important to acknowledge that not all bacteria display this behavior: the suicidal release of toxins is known from several bacterial species [1–5], but it is not the norm across species. Moreover, in the case of *E. coli*, we only expect these levels of cell lysis to occur when a strain is targeted by DNA-damaging toxins that allow it to detect the incoming attack and respond with its own colicin. Some colicins have other mechanisms of action, including pore-forming colicins that are "silent" toxins that can kill cells without activating the SOS response or toxin release in a competitor (Figure 1C) [11, 22]. Nevertheless, the high frequency of cell suicide at the interface between DNase-producing strains is striking and represents one of the most extreme social phenotypes documented to date in bacteria or elsewhere.

When will mass cell suicide be favored by natural selection? Because it occurs in cells that are going to die anyway, lysis of these cells is expected to be evolutionarily beneficial whenever toxin production is beneficial to a genotype. Previous work has addressed the costs and benefits of toxin release between DNase colicin-producing strains [11, 22]. This suggested that high levels of toxin production are most favored when a strain is numerically dominant and able to mount a response that will rapidly overcome a competitor [11]. A twist in the case of colicin production is that the genes for the behavior are carried on plasmids that, under some conditions, can be subject to horizontal transfer between strains via conjugation [42]. Gene mobility has the potential to lead to additional complexities where natural selection on the plasmids differs from that of their hosts. However, colicin plasmids cannot always conjugate (as in our experiments), and suicidal cell behaviors are encoded in the chromosome in species like *Pseudomonas aeruginosa* [3]. The potential for conjugation, therefore, is not a requirement for the evolution of toxin release via cell lysis.

The evolution of self-lysis is also known from the phenomenon of abortive phage infection in bacteria [43–45]. Here, infected cells have evolved to lyse rather than pass on the infection to clonemates, and the great majority of cells will undergo lysis if enough phage are added to a culture [46]. However, in terms of function, much more similar are examples from the social insects. Massive suicidal attacks are known from several insect species (Figure 4C) where they are performed by the old workers that have the lowest reproductive and helping potential in the colony [6–9]. We see a clear parallel in our experiments where the highest levels of cell suicide occur under conditions where the cells are going to die anyway from exposure to the toxin. These cells are no longer able to divide, and, as in the social insect examples, this loss of reproductive potential is associated with the expression of the attack behavior. This makes the fitness costs associated with this extreme behavior close to zero and, so long as there are some clonemates in the vicinity that can benefit, the behavior can be favored by natural selection (more specifically kin selection [39, 47]). Our work suggests a strong evolutionary convergence in the sociality of bacteria and that of the social insects. In both, examples of mass self-sacrifice are seen in defense of the colony and, in both, these behaviors can be explained by low personal fitness costs combined with benefits to kin.

STAR★METHODS

Detailed methods are provided in the online version of this paper and include the following:

- KEY RESOURCES TABLE
- RESOURCE AVAILABILITY
  - Lead Contact
  - Materials Availability
  - Data and Code Availability
- EXPERIMENTAL MODEL AND SUBJECT DETAILS
  - Bacterial growth conditions
  - Construction of bacterial strains
- METHOD DETAILS
  - Whole-colony competitions
  - Supernatant exposure assays
  - Colony monolayer imaging
  - 3D colony imaging
- QUANTIFICATION AND STATISTICAL ANALYSIS

SUPPLEMENTAL INFORMATION

Supplemental Information can be found online at https://doi.org/10.1016/j.cub.2020.05.007.

ACKNOWLEDGMENTS

We thank Despoina Mavridou and Diego Gonzalez for strains and plasmids and Michael Bentley, Rolf Kümmerli, Despoina Mavridou, Jacob Palmer, Will Smith, and Sofia van Moorsel for comments on the manuscript. We are also grateful to three anonymous reviewers for their constructive input. E.T.G. is funded by a Postdoc Mobility Fellowship from the Swiss National Science Foundation (project no. P2ZHP3_174751 and P400PB_183878). K.R.F. is funded by European Research Council Grant 787932 and Wellcome Trust Investigator award 209397/Z/17/Z.

AUTHOR CONTRIBUTIONS

E.T.G. and K.R.F. designed experiments; E.T.G. performed experiments; E.T.G. performed statistical analyses; E.T.G. and K.R.F. interpreted data; E.T.G. and K.R.F. wrote the manuscript.

DECLARATION OF INTERESTS

The authors declare no competing interests.

Received: February 28, 2020
Revised: March 31, 2020
Accepted: May 4, 2020
Published: June 4, 2020

REFERENCES

1. Cascales, E., Buchanan, S.K., Duché, D., Kleanthous, C., Lloubès, R., Postle, K., et al. (2007). Colicin Biology. Microbiol. Mol. Biol. Rev. *71*, 158–229.
2. Pugsley, A.P., Goldzahl, N., and Barker, R.M. (1985). Colicin E2 production and release by Escherichia coli K12 and other Enterobacteriaceae. J. Gen. Microbiol. *131*, 2673–2686.
3. Michel-Briand, Y., and Baysse, C. (2002). The pyocins of *Pseudomonas aeruginosa*. Biochimie *84*, 499–510.
4. Šmajs, D., Pilsl, H., and Braun, V. (1997). Colicin U, a novel colicin produced by Shigella boydii. J. Bacteriol. *179*, 4919–4928.
5. Riley, M.A., Pinou, T., Wertz, J.E., Tan, Y., and Valletta, C.M. (2001). Molecular characterization of the klebicin B plasmid of Klebsiella pneumoniae. Plasmid *45*, 209–221.
6. Šobotník, J., Bourguignon, T., Hanus, R., Demianová, Z., Pytelková, J., Mareš, M., Foltynová, P., Preisler, J., Cvačka, J., Krasulová, J., and Roisin, Y. (2012). Explosive backpacks in old termite workers. Science *337*, 436.
7. Laciny, A., Zettel, H., Kopchinskiy, A., Pretzer, C., Pal, A., Salim, K.A., Rahimi, M.J., Hoenigsberger, M., Lim, L., Jaitrong, W., and Druzhinina, I.S. (2018). *Colobopsis explodens* sp. n., model species for studies on "exploding ants" (Hymenoptera, Formicidae), with biological notes and first illustrations of males of the *Colobopsis cylindrica* group. ZooKeys *2018*, 1–40.
8. Nouvian, M., Reinhard, J., and Giurfa, M. (2016). The defensive response of the honeybee Apis mellifera. J. Exp. Biol. *219*, 3505–3517.
9. Shorter, J.R., and Rueppell, O. (2012). A review on self-destructive defense behaviors in social insects. Insectes Soc. *59*, 1–10.
10. Pugsley, A.P., and Rosenbusch, J.P. (1981). Release of colicin E2 from Escherichia coli. J. Bacteriol. *147*, 186–192.
11. Mavridou, D.A.I., Gonzalez, D., Kim, W., West, S.A., and Foster, K.R. (2018). Bacteria Use Collective Behavior to Generate Diverse Combat Strategies. Curr. Biol. *28*, 345–355.
12. Mulec, J., Podlesek, Z., Mrak, P., Kopitar, A., Ihan, A., and Žgur-Bertok, D. (2003). A cka-gfp transcriptional fusion reveals that the colicin K activity gene is induced in only 3 percent of the population. J. Bacteriol. *185*, 654–659.
13. Kamenšek, S., Podlesek, Z., Gillor, O., and Žgur-Bertok, D. (2010). Genes regulated by the Escherichia coli SOS repressor LexA exhibit heterogeneous expression. BMC Microbiol. *10*, 283.
14. Bayramoglu, B., Toubiana, D., van Vliet, S., Inglis, R.F., Shnerb, N., and Gillor, O. (2017). Bet-hedging in bacteriocin producing Escherichia coli populations: the single cell perspective. Sci. Rep. *7*, 42068.
15. Ozeki, H., Stocker, B.A.D., and De Margerie, H. (1959). Production of colicine by single bacteria. Nature *184*, 337–339.
16. Ghazaryan, L., Tonoyan, L., Ashhab, A.A., Soares, M.I.M., and Gillor, O. (2014). The role of stress in colicin regulation. Arch. Microbiol. *196*, 753–764.
17. Ghazaryan, L., Soares, M.I.M., and Gillor, O. (2014). Auto-regulation of DNA degrading bacteriocins: molecular and ecological aspects. Antonie van Leeuwenhoek *105*, 823–834.
18. Gillor, O., Vriezen, J.A.C., and Riley, M.A. (2008). The role of SOS boxes in enteric bacteriocin regulation. Microbiology *154*, 1783–1792.
19. Žgur-Bertok, D. (2012). Regulating colicin synthesis to cope with stress and lethality of colicin production. Biochem. Soc. Trans. *40*, 1507–1511.
20. Mader, A., von Bronk, B., Ewald, B., Kesel, S., Schnetz, K., Frey, E., and Opitz, M. (2015). Amount of colicin release in Escherichia coli is regulated by lysis gene expression of the colicin E2 operon. PLoS ONE *10*, e0119124.
21. Pugsley, A.P., and Schwartz, M. (1983). A genetic approach to the study of mitomycin-induced lysis of Escherichia coli K-12 strains which produce colicin E2. Mol. Gen. Genet. *190*, 366–372.
22. Gonzalez, D., Sabnis, A., Foster, K.R., and Mavridou, D.A.I. (2018). Costs and benefits of provocation in bacterial warfare. Proc. Natl. Acad. Sci. USA *115*, 7593–7598.
23. Cornforth, D.M., and Foster, K.R. (2013). Competition sensing: the social side of bacterial stress responses. Nat. Rev. Microbiol. *11*, 285–293.
24. Kuhar, I., and Žgur-Bertok, D. (1999). Transcription regulation of the colicin K cka gene reveals induction of colicin synthesis by differential responses to environmental signals. J. Bacteriol. *181*, 7373–7380.
25. Majeed, H., Gillor, O., Kerr, B., and Riley, M.A. (2011). Competitive interactions in Escherichia coli populations: the role of bacteriocins. ISME J. *5*, 71–81.
26. Pugsley, A.P., and Schwartz, M. (1984). Colicin E2 release: lysis, leakage or secretion? Possible role of a phospholipase. EMBO J. *3*, 2393–2397.
27. Götz, A., Lechner, M., Mader, A., von Bronk, B., Frey, E., and Opitz, M. (2018). CsrA and its regulators control the time-point of ColicinE2 release in Escherichia coli. Sci. Rep. *8*, 6537.
28. Yang, T.Y., Sung, Y.M., Lei, G.S., Romeo, T., and Chak, K.F. (2010). Posttranscriptional repression of the cel gene of the ColE7 operon by the RNA-binding protein CsrA of Escherichia coli. Nucleic Acids Res. *38*, 3936–3951.
29. Chang, H.W., Yang, T.Y., Lei, G.S., and Chak, K.F. (2013). A novel endogenous induction of ColE7 expression in a csrA mutant of Escherichia coli. Curr. Microbiol. *66*, 392–397.
30. Lechner, M., Schwarz, M., Opitz, M., and Frey, E. (2016). Hierarchical Post-transcriptional Regulation of Colicin E2 Expression in Escherichia coli. PLoS Comput. Biol. *12*, e1005243.
31. Berney, M., Hammes, F., Bosshard, F., Weilenmann, H.U., and Egli, T. (2007). Assessment and interpretation of bacterial viability by using the LIVE/DEAD BacLight Kit in combination with flow cytometry. Appl. Environ. Microbiol. *73*, 3283–3290.
32. Nadell, C.D., Drescher, K., and Foster, K.R. (2016). Spatial structure, cooperation and competition in biofilms. Nat. Rev. Microbiol. *14*, 589–600.
33. Ghoul, M., and Mitri, S. (2016). The Ecology and Evolution of Microbial Competition. Trends Microbiol. *24*, 833–845.
34. Moffett, M. (2013). Comparative canopy biology and the structure of ecosystems. In Treetops at Risk: Challenges of Global Canopy Ecology and Conservation, M. Lowman, S. Devy, and T. Ganesh, eds. (Springer), pp. 13–54.
35. Flemming, H.C., and Wuertz, S. (2019). Bacteria and archaea on Earth and their abundance in biofilms. Nat. Rev. Microbiol. *17*, 247–260.
36. Mark Welch, J.L., Hasegawa, Y., McNulty, N.P., Gordon, J.I., and Borisy, G.G. (2017). Spatial organization of a model 15-member human gut microbiota established in gnotobiotic mice. Proc. Natl. Acad. Sci. USA *114*, E9105–E9114.
37. Tropini, C., Earle, K.A., Huang, K.C., and Sonnenburg, J.L. (2017). The Gut Microbiome: Connecting Spatial Organization to Function. Cell Host Microbe *21*, 433–442.
38. Pugsley, A.P. (1983). Autoinduced synthesis of colicin E2. Mol. Gen. Genet. *190*, 379–383.
39. Hamilton, W.D. (1964). The genetical evolution of social behaviour. I. J. Theor. Biol. *7*, 1–16.
40. Granato, E.T., Meiller-Legrand, T.A., Foster, K.R., and Foster, K.R. (2019). The evolution and ecology of bacterial warfare. Curr. Biol. *29*, R521–R537.

41. Gardner, A., West, S.A., and Buckling, A. (2004). Bacteriocins, spite and virulence. Proc. Biol. Sci. *271*, 1529–1535.

42. Finnegan, J., and Sherratt, D. (1982). Plasmid ColE1 conjugal mobility: the nature of bom, a region required in cis for transfer. Mol. Gen. Genet. *185*, 344–351.

43. Fineran, P.C., Blower, T.R., Foulds, I.J., Humphreys, D.P., Lilley, K.S., and Salmond, G.P.C. (2009). The phage abortive infection system, ToxIN, functions as a protein-RNA toxin-antitoxin pair. Proc. Natl. Acad. Sci. USA *106*, 894–899.

44. Smith, H.S., Pizer, L.I., Pylkas, L., and Lederberg, S. (1969). Abortive infection of Shigella dysenteriae P2 by T2 bacteriophage. J. Virol. *4*, 162–168.

45. Chopin, M.C., Chopin, A., and Bidnenko, E. (2005). Phage abortive infection in lactococci: variations on a theme. Curr. Opin. Microbiol. *8*, 473–479.

46. Refardt, D., Bergmiller, T., and Kümmerli, R. (2013). Altruism can evolve when relatedness is low: evidence from bacteria committing suicide upon phage infection. Proc. Biol. Sci. *280*, 20123035.

47. Mitri, S., and Foster, K.R. (2013). The genotypic view of social interactions in microbial communities. Annu. Rev. Genet. *47*, 247–273.

48. R Development Core Team (2018). R: A language and environment for statistical computing (R Foundation for Statistical Computing).

49. Schindelin, J., Arganda-Carreras, I., Frise, E., Kaynig, V., Longair, M., Pietzsch, T., Preibisch, S., Rueden, C., Saalfeld, S., Schmid, B., et al. (2012). Fiji: an open-source platform for biological-image analysis. Nat. Methods *9*, 676–682.

50. Meacock, O. (2020). FAST 0.9.1. Available at. https://doi.org/10.5281/zenodo.3630642.

51. Hartmann, R., Jeckel, H., Jelli, E., Singh, P.K., Vaidya, S., Bayer, M., Vidakovic, L., Díaz-Pascual, F., Fong, J.C.N., Dragoš, A., et al. (2019). BiofilmQ, a software tool for quantitative image analysis of microbial biofilm communities. bioRxiv. https://doi.org/10.1101/735423.

52. Wickham, H., François, R., Henry, L., and Müller, K. (2018). dplyr: A Grammar of Data Manipulation. R package version 0.7.6.

53. Hope, R.M. (2013). Rmisc: Ryan Miscellaneous. R package version 1.5.

54. Valero-Mora, P.M. (2010). ggplot2: Elegant Graphics for Data Analysis. J. Stat. Softw. *35*, 212.

55. Wilke, C.O. (2019). cowplot: Streamlined Plot Theme and Plot Annotations for "ggplot2". R package version 0.9.4.

56. Bates, D., Mächler, M., Bolker, B., and Walker, S. (2014). Fitting Linear Mixed-Effects Models Using lme4. Journal of Statistical Software *67*, 1–48.

STAR★METHODS

KEY RESOURCES TABLE

REAGENT or RESOURCE	SOURCE	IDENTIFIER
Bacterial and Virus Strains		
BZB1011 WT (wildtype): W3110, gyrA, Str^R	[21]	N/A
WT *gfp*: BZB1011 Tn7::P*max*::*gfp*	[11]	N/A
ColE2 *gfp*: BZB1011 Tn7::P*max*::*gfp* pColE2	[11]	N/A
WT pUA66-P*colE2*::*gfp*: BZB1011 pUA66-P*colE2*::*gfp*, Kan^R	This study	N/A
ColE2 pUA66-P*colE2*::*gfp*: BZB1011 pColE2 pUA66-P*colE2*::*gfp*, Kan^R	[11]	N/A
ColE1: BZB1011 pColE1	[22]	N/A
ColE8: BZB1011 pColE8	[11]	N/A
Chemicals, Peptides, and Recombinant Proteins		
All antibiotics for bacterial culture	Sigma-Aldrich	N/A
Propidium iodide solution	Sigma-Aldrich	P4864-10ML
Deposited Data		
Raw and analyzed data	This study	https://doi.org/10.5281/zenodo.3758060
Recombinant DNA		
pColE1 (pColE1-K53): Colicin E1 natural plasmid	[16]	N/A
pColE2 (pColE2-P9): Colicin E2 natural plasmid	[16]	N/A
pColE8 (pColE8-J): Colicin E8 natural plasmid	[16]	N/A
pUA66-P*colE2*::*gfp*: GFPmut3 transcribed from the *colE2* promoter, Kan^R	[11]	N/A
Software and Algorithms		
RStudio version 1.1.414	[48]	http://www.R-project.org
Zen Blue	Zeiss	RRID:SCR_013672
Zen Black	Zeiss	RRID:SCR_013672
FIJI	[49]	http://fiji.sc; RRID:SCR_002285
FAST v0.9.1	Oliver Meacock [50],	https://doi.org/10.5281/zenodo.3630642

RESOURCE AVAILABILITY

Lead Contact

Further information and requests for resources and reagents should be directed to and will be fulfilled by the Lead Contact, Dr. Kevin R. Foster (kevin.foster@zoo.ox.ac.uk).

Materials Availability

This study did not generate new unique reagents.

CellPress
OPEN ACCESS

Data and Code Availability

Original data generated in this study have been deposited to Zenodo: https://doi.org/10.5281/zenodo.3758060.

EXPERIMENTAL MODEL AND SUBJECT DETAILS

Bacterial growth conditions

Unless otherwise indicated, all *E. coli* BZB1011 strains were grown overnight in 5 mL LB medium (per L: 10 g Tryptone, 10 g NaCl, 5g Yeast Extract) in 15 mL polypropylene tubes at 37°C with agitation (220 rpm). When necessary, the medium was supplemented with kanamycin (50 μg/mL). All experiments were carried out at 37°C. For time-lapse microscopy experiments, samples were kept at 37°C at all times using a custom-built incubation chamber. Whole-colony competitions (Figures 1B–1D) were carried out on 0.8% w/v LB Agar. Supernatant exposure assays (Figures 2, 4A, and S1) were carried out on 0.8% w/v LB Agarose supplemented with 1 μg/mL propidium iodide (PI; Sigma-Aldrich). Colony monolayer assays (Figures 3A and 3B) and 3D colony imaging experiments (Figures 3C, 3D, and S2) were carried out on 0.8% w/v LB Agar supplemented with 1 μg/mL PI.

Construction of bacterial strains

For the generation of the BZB1011 WT pUA66-P*colE2*::*gfp* strain, BZB1011 WT cells were transformed with the reporter plasmid pUA66-P*colE2*::*gfp* [11] via electroporation and selected on 50 μg/mL kanamycin at 37°C. All plasmids and strains used in this study are listed in the Key Resources Table. Throughout the manuscript, we refer to the strain observed with microscopy as the "focal" strain to aid in distinguishing it from its competitor, which constitutes the treatment the focal strain is subjected to.

METHOD DETAILS

Whole-colony competitions

To monitor the response of a colicin E2 producing strain to foreign colicins with different cellular targets (Figures 1B–1D), cells were grown to exponential phase, washed twice with LB medium, and resuspended and normalized in LB medium to an optical density at 600 nm (OD600) of 1.0 for the competitor (ColE8, ColE1, or WT as a negative control) and a 10^{-3} dilution of OD600 = 1.0 for the focal strain (ColE2 pUA66-P*colE2*::*gfp*). Per strain combination, three 5 μL spots of bacterial suspension were then spotted next to each other onto 0.8% w/v LB agar plates, resulting in a distance of approximately 0.5 cm between the respective spot edges. Plates were then incubated at 37°C for 12 hours. After incubation, bright field and GFPmut3-fluorescence (ex: 500 nm|em: 513 nm) images of whole colonies were acquired using a Zeiss Plan-Apochromat Z 0.5 × objective on a Zeiss AxioZoom.V16 stereomicroscope with ZEN Blue software (version 1.1.2.0). Colony edges of the same colonies were then surface-imaged using a Zeiss EC Epiplan-Neofluar 50x air objective (NA = 0.8) on a Zeiss LSM880 confocal laser scanning unit in regular confocal mode, using ZEN Black software (version 14.0.18.201). Images were analyzed using *FIJI* [49], and Figures 1B–1D show representative images of three biological replicates.

Supernatant exposure assays

To record time courses of cells reacting to sterile supernatant of a competitor, cells of the competitor strain (ColE8, ColE2, or WT as a control) were grown overnight for 16 hours, and 1 mL of bacterial suspension was centrifuged for 5 min at 17500*g to sediment cells. The resulting supernatant was sterile-filtered using a 0.2 μm syringe filter (Sartorius) and then serially diluted in sterile saline (0.8% NaCl in ddH_2O). 3 μL of undiluted or diluted sterile supernatant were then pipetted onto small circular cut-outs (diameter 5 mm, height 2 mm) of 0.8% w/v LB agarose + PI and left to dry and diffuse in the agarose pad for 1 hour at room temperature. In parallel, cells of the focal strain (ColE2 pUA66-P*colE2*::*gfp*, or WT pUA66-P*colE2*::*gfp*) were grown to exponential phase (OD600 ~0.2), washed twice with LB medium, resuspended, and then diluted 1:50 with LB medium. 1 μL of this diluted bacterial suspension was then spotted onto the agarose pads infused with supernatant and left to dry for 10 minutes at room temperature. Pads were then placed onto a glass slide and covered with a 22x22mm n° 1.5 coverslip so that the cells were sandwiched between the agarose and the coverslip. The sides of the coverslip were sealed with *Glisseal-HV* laboratory grease (VWR) to avoid desiccation. The sample was then moved to the microscope and imaged immediately. Time-lapse fluorescence microscopy was performed using a Zeiss Axio Observer inverted microscope with a Zeiss Plan-Apochromat 63x oil immersion objective (NA = 1.4) and ZEN Blue software (version 1.1.2.0). Exposure times were 156 ms for phase contrast, 100 ms for GFPmut3 (ex: 500 nm|em: 513 nm), and 50 ms for PI (ex: 493 nm|em: 636 nm). Images were acquired every 5 minutes for 6 hours. Image analysis and cell tracking was carried out with *FIJI* [49] and *FAST* [50]. For the data presented in Figures 2D and 4A, three or four biological replicates were carried out for each supernatant concentration and strain combination, and for each replicate at least three fields of view were recorded at each time point. A total number of n = 7985 and 3266 cells were tracked for the two focal strains ColE2 pUA66-P*colE2*::*gfp* and WT pUA66-P*colE2*::*gfp*, respectively. Cells that failed to divide during the six hour observation time were counted as dead, and cells that started dividing were counted as such. The vast majority of cells scored as "dividing" started dividing within the first hour of observation and kept dividing continuously throughout the observation period. In treatments where cells were exposed to supernatants containing DNase toxins, these are the cells that were able to overcome DNA damage and sustain division. Cells that exceeded a PI-specific fluorescence value over a threshold of 3x the background value during the observation period were categorized as PI-positive, cells below that value as PI-negative. The data presented in Figure 2C is a subset of the data presented in Figure 2D and shows background-corrected

and GFP- and PI-specific fluorescence values for all tracked ColE2 pUA66-P*colE2*::*gfp* cells in one field of view exposed to 1% ColE8 supernatant. For visualization purposes, time-series values were synchronized with respect to time-to-lysis in each cell.

Colony monolayer imaging

To image colony monolayers of a focal strain reacting to a nearby competitor, cells were grown to exponential phase, washed twice with LB medium, and resuspended and normalized in LB medium to an OD600 of 1.0 for the competitor (ColE8) and 0.05 for the focal strain (ColE2 pUA66-P*colE2*::*gfp*, or WT pUA66-P*colE2*::*gfp*). 5 μL of bacterial suspension per strain were then spotted next to each other onto 0.8% w/v LB agar + PI plates, resulting in a distance of approximately 1.0 mm between the spot edges. After drying for 10 minutes at room temperature, a ca. 2x2 cm section of agar around a pair of seeded spots (competitor and focal strain) was then cut out using a scalpel, and placed onto a 5 cm diameter glass bottom Petri dish with a 3-cm diameter uncoated n°1.5 glass window (MatTek Corporation), which was then inverted to allow immediate imaging of the spots through air. Time-lapse imaging of the spot edge at the interface between the competitor and the focal strain was then performed using a Zeiss Axio Observer inverted microscope with a Zeiss Plan-Apochromat 20x objective (NA = 0.8) and ZEN Blue software (version 2.6.76.00000). Exposure times were 2.72 ms for bright-field, 20 ms for GFPmut3 (ex: 500 nm|em: 513 nm), and 80 ms for PI (ex: 493 nm | em: 636 nm). Images were acquired every five minutes for 6 hours, and image analysis and cell tracking was carried out with *FIJI* [49] and *FAST* [50]. For the data presented in Figure 3B, three biological replicates were carried out for each strain, and for each replicate six fields of view at different distances from the colony edge were recorded at each time point. A combined total number of n = 3420 cells were successfully tracked over ≥ 4 hours for the two focal strains ColE2 pUA66-P*colE2*::*gfp* and WT pUA66-P*colE2*::*gfp*. Cells that exceeded a PI-specific fluorescence value over a threshold of 3x the background value during the observation period were categorized as PI-positive, cells below that value as PI-negative. Self-lysis frequencies were determined by calculating the frequency of PI-positive cell tracks relative to the number of cells tracked in that position.

3D colony imaging

To image three-dimensional colonies of a focal strain reacting to a nearby competitor, cells were grown to exponential phase, washed twice with LB medium, and resuspended and normalized in LB medium to an OD600 of 1.0 for both the competitor (ColE8) and the focal strain (ColE2 *gfp*, or WT *gfp*). 5 μL of bacterial suspension per strain were then spotted next to each other onto a glass bottom Petri dish filled with 8 mL 0.8% w/v LB agar + PI, resulting in a distance of approximately 1.0 mm between the spot edges. After drying for ten minutes at room temperature, the plate was inverted to allow for immediate imaging of the colonies through air. Time-lapse confocal imaging of the focal strain colony edge facing the competitor was then carried out using a Zeiss EC Epiplan-Neofluar 50x air objective (NA = 0.8) on a Zeiss LSM880 confocal laser scanning unit in *Airyscan* mode, using ZEN Black software (version 14.0.18.201). Three-dimensional z stacks at 0.36 μm intervals were then acquired every hour for 8 hours. Airyscan-processed images (processing strength = 6.0) were rendered in 3D using ZEN Blue (version 2.3.69.1018) and analyzed using *BiofilmQ* v0.1.4 [51]. For the data presented in Figures 3D and S2, three or four biological replicates were carried out for each strain, and for each replicate six fields of view at different distances from the colony edge were recorded at each time point. Self-lysis in each field of view was quantified by determining the volume of biomass exhibiting PI-specific fluorescence (indicating self-lysis in strain ColE2 *gfp*) relative to the total biomass. Total biomass was calculated by determining the volume of biomass exhibiting GFP-specific fluorescence (indicating either live cells or dead cells that did not self-lyse) plus volume of the biomass exhibiting PI-specific fluorescence.

QUANTIFICATION AND STATISTICAL ANALYSIS

Statistical analysis and data visualization were performed using RStudio version 1.1.414 [48] and packages *dplyr* [52], *Rmisc* [53], *ggplot2* [54], *cowplot* [55] and *lme4* [56]. For all statistical tests, the significance level α was set to 0.01. To test whether the frequency of PI-positive cells depended on supernatant concentrations (Figures 2D and 4A), we used non-parametric Kruskal-Wallis tests. To test whether ColE2 and wild-type cells differed in their frequencies of PI-positive cells when reacting to competitor supernatant in thin colonies (Figure 3B) and three-dimensional colonies (Figures 3D and S2), we used linear models as implemented in the *lme4* package [56]. Statistical details (sample size n, syntax for linear models) for each experiment are provided in the respective Figure legends as well as in the STAR Methods section.

Current Biology, Volume *30*

Supplemental Information

The Evolution of Mass Cell Suicide in Bacterial Warfare

Elisa T. Granato and Kevin R. Foster

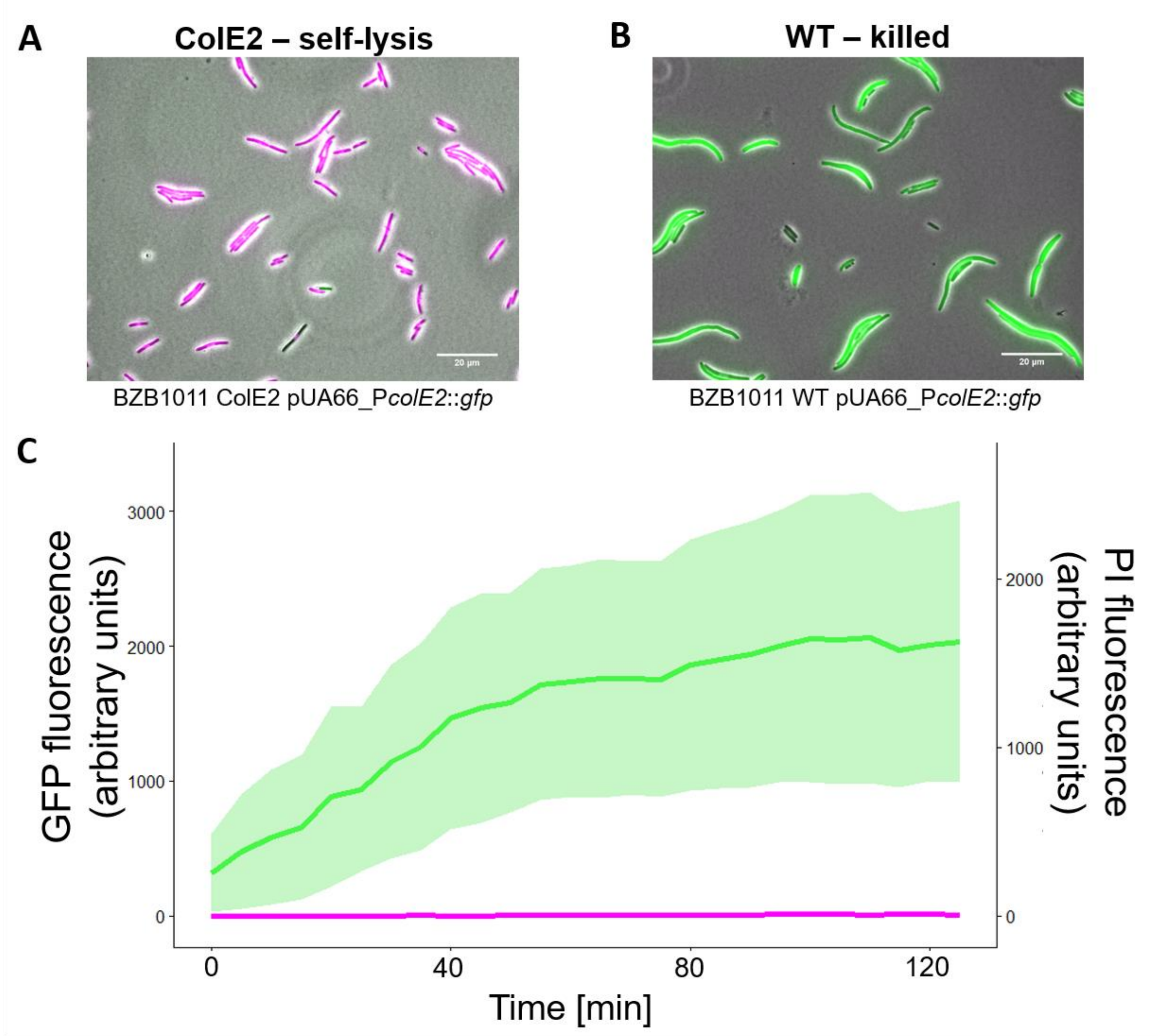

Figure S1. *E. coli* cells unable to self-lyse do not exhibit elevated PI-fluorescence. See also Figure 2. **(A+B)** Representative images of ColE2 pUA66-P*colE2*::*gfp* cells having undergone self-lysis **(A)** or WT pUA66-P*colE2*::*gfp* cells having been killed by a foreign DNase colicin **(B)**. Phase-contrast channel, GFP channel and propidium iodide (PI) channel are overlaid in all images. GFP signal indicates colicin promoter activation. PI signal indicates membrane permeabilization, i.e. self-lysis. Absence of PI signal in a non-dividing, dead cell is indicative of an intact membrane and hence killing by the action of the foreign DNase colicin. Image (A) represents the final timepoint of the dataset shown in Figure 2C. Image shown in (B) represents the final timepoint of the dataset shown in (C) here. Scale bars, 20 µm. **(C)** Fluorescence signals in WT cells responding to colicin E8. WT pUA66-P*colE2*::*gfp* cells were exposed to a 1% dilution of supernatant of a colicin E8-producing strain and imaged for up to 6 hours. Individual cell fluorescence tracks are shown for the GFP channel (green) and PI channel (magenta). Thick lines and shaded areas indicate the mean and standard deviation across n = 31 tracked cells in the same field of view. See Video S4.

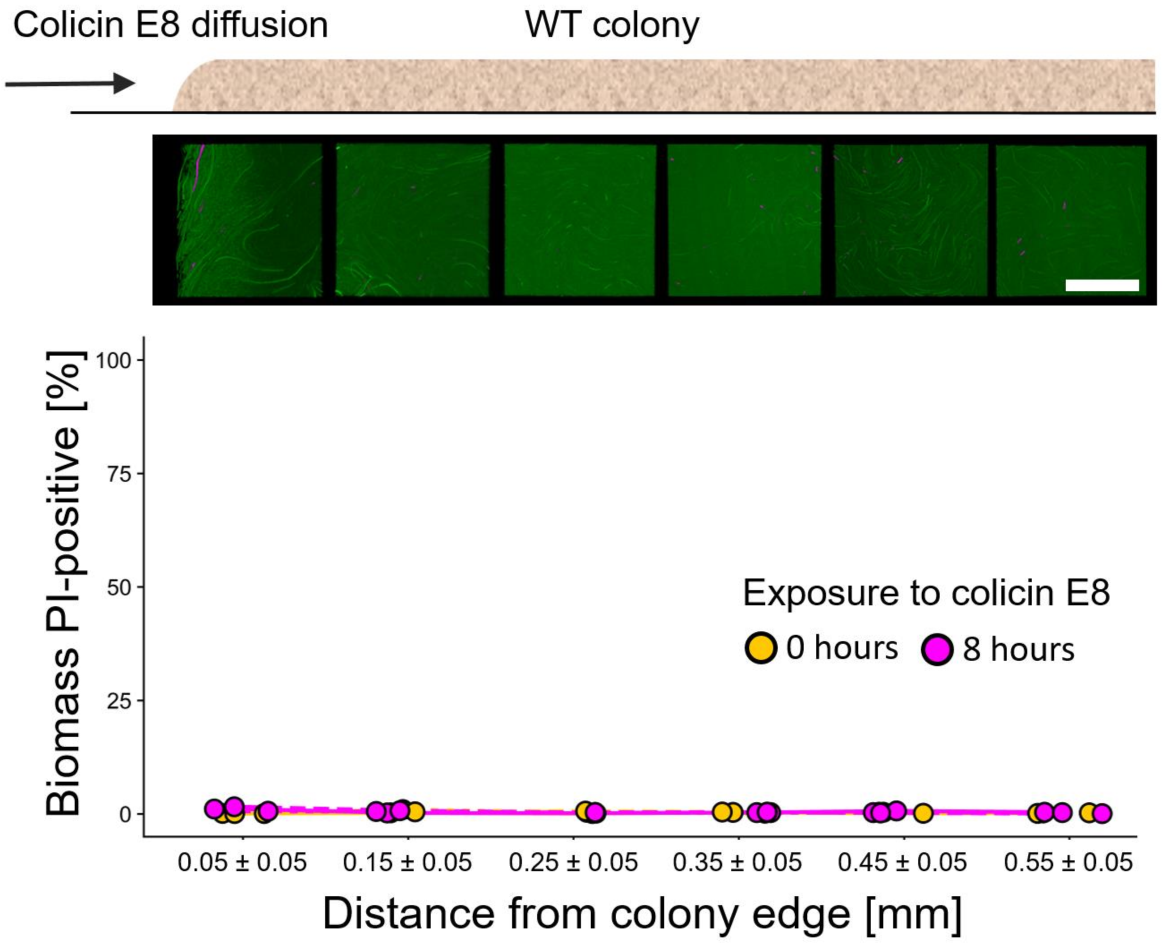

Figure S2. Three-dimensional *E. coli* colonies unable to self-lyse do not exhibit elevated PI-fluorescence. See also Figure 3. Quantification of PI-specific fluorescence in three-dimensional colonies exposed to colicin E8 produced by a nearby colony. Cells of a focal strain producing GFP constitutively but not capable of producing colicin E2 or self-lysing (WT *gfp*) were grown in a three-dimensional colony on nutrient medium supplement with propidium iodide (PI, 1µg/mL) next to a strain producing colicin E8. The focal colony was imaged for 8 hours using time-lapse 3D confocal microscopy at six locations situated at different distances from the colony edge facing the competitor. The proportion of PI-specific fluorescence at each location was quantified by determining the volume of biomass exhibiting PI-specific fluorescence relative to the total biomass, after zero or 8 hours of exposure to colicin E8. Total biomass was calculated by determining the volume of biomass exhibiting GFP-specific fluorescence (indicating either live cells or dead cells) plus biomass of those exhibiting PI-specific fluorescence. Line-types indicate three independent biological replicates. We did not detect a statistically significant increase in PI-specific fluorescence over the observation period at any location (linear model: percent.positive ~ timepoint; $F(1,4) \leq 11.89$ for all distances; $p > .01$). Images above each distance point show 3D renderings of confocal images of the same replicate colony acquired at the respective position after 8 hours of exposure, viewed from above. Scale bar, 50 µm.
